# Perceptual restoration of degraded speech: The effects of linguistic structure

**DOI:** 10.3758/s13414-025-03128-0

**Published:** 2025-08-04

**Authors:** Mako Ishida, Takayuki Arai, Makio Kashino

**Affiliations:** 1 Faculty of Science and Technology, Department of Foreign Language and Liberal Arts, Keio University, 4 Chome-1-1 Hiyoshi, Kohoku Ward, Yokohama, Kanagawa 223-8521 Japan; 2 Information and Communication Sciences, Sophia University, Tokyo, Japan; 3 NTT Communication Science Laboratories, Atsugi, Japan

**Keywords:** Perceptual restoration, Speech perception, Linguistic structure, Mora, Syllable, Locally time-reversed speech, Modulation-filtered speech

## Abstract

Listeners can understand speech even when its temporal structure is acoustically distorted. Ishida et al. (*Frontiers in Psychology, 9*, 1749, 2018) reported that native English speakers could comprehend English sentences using two types of temporal distortions: (1) speech signals divided into equally timed segments, with each segment reversed in time (locally time-reversed speech), and (2) speech signals with reduced modulation-frequency components shaping the amplitude envelope (modulation-filtered speech). While the results showed a similar pattern of intelligibility decline across these two conditions in English (a syllable-oriented language with consonant clusters) when degradation increased in six steps, it remained unclear whether this pattern holds in a linguistically distinct language like Japanese (a mora-oriented language with CV and V as basic linguistic units). The current study investigates how native Japanese speakers comprehend Japanese sentences under the same temporal distortions. In Experiment 1, participants listened to locally time-reversed Japanese sentences with segment intervals reversed at 10 ms, 30 ms, 50 ms, 70 ms, 90 ms, and 110 ms. In Experiment 2, the same participants listened to modulation-filtered Japanese sentences, where the modulation frequency components were low-pass filtered at cut-off frequencies of 32 Hz, 16 Hz, 8 Hz, 4 Hz, 2 Hz, and 1 Hz. Results showed that the intelligibility of locally time-reversed and modulation-filtered Japanese sentences decreased as distortion increased, with longer reversed segment lengths and lower cut-off frequencies. However, the patterns of intelligibility degradation in Japanese differed significantly from those in English. Thus, perceptual restoration may function differently depending on the basic linguistic units (mora vs. syllable).

**Supplementary Information:**

The online version contains supplementary material available at 10.3758/s13414-025-03128-0.

## Introduction

Speech comprehension involves the process in which listeners perceptually restore a missing or degraded portion of speech to make sense of what is being said (perceptual restoration). Warren (1970) reported that people could understand speech even when part of the speech signal was replaced by noise using the sentence: “The state governors met with their respective legislatures convening in the capital city.” When the first phoneme “s” in “legislatures” was replaced by extraneous sounds such as white noise, buzzes, or tones, listeners could still understand the sentence without noticing the missing part of speech. Even when the listeners were told which sound was missing, they still perceived the continuity of the speech, although the gap was noticeable if left silent. In fact, perceptual restoration follows the masking potential rule (Bashford et al., 1992; Houtgast, 1972; Kashino, 2006; Kashino & Warren, 1996; Warren, 1970; Warren & Warren, 1970; Warren et al., 1972). That is, people can perceptually restore the missing portion of speech: (1) when the replacing sound is as loud as or louder than the original phoneme (Warren, 1970; Warren & Warren, 1970); (2) when the replaced and replacing sound have the same center frequency (Bashford & Warren, 1987; Warren et al., 1972; Warren, 1984); and (3) when the replaced and replacing sounds are acoustically similar (Samuel, 1981a, 1981b) – for example, fricatives and stops are perceptually restored most effectively when replaced by white noise but least when replaced by pure tones, while vowels are perceptually restored most effectively by pure tones but least by white noise. Additionally, perceptual restoration is influenced by linguistic factors such as (a) coarticulation (Kashino, 1990; Kashino et al., 1992), (b) contextual information (Ganong, 1980; Leonard et al., 2016; Nagaraj & Magimairaj, 2017; Samuel, 1981a, 1981b, 1996; Warren & Obusek, 1971; Warren & Sherman, 1974), and (c) listeners’ proficiency in the target language (Ishida & Arai, 2016; Kashino & Craig, 1994; Warren & Obusek, 1971; Warren & Warren, 1970). These studies of perceptual restoration have shown that listening is a process of integrating both bottom-up acoustic cues and top-down linguistic cues to comprehend degraded speech, which is an essential skill in daily life where listeners are surrounded by random noise and acoustic artifacts.

Perceptual restoration studies were later conducted on a larger scale by degrading the entire speech signal (rather than individual phonemes or speech segments), using locally time-reversed speech where the speech signal was divided into fixed-duration segments from the onset of speech, with each segment reversed in time. Saberi and Perrott (1999) reported that people could perceptually restore locally time-reversed spoken sentences by integrating the dispersed information in time, and the intelligibility of locally time-reversed speech gradually decreased as the length of each reversed segment increased. Subsequent studies also confirmed this general trend of intelligibility decline (Grataloup et al., 2009; Greenberg & Arai, 2001; Ishida, 2021; Ishida et al., 2016, 2018; Kiss et al., 2008; Magrin-Chagnolleau et al., 2002; Remez et al., 2013; Stilp et al., 2010; Ueda et al., 2017), although the methodologies (e.g., subjective intelligibility reporting vs. transcription) and the units of measurement assessing intelligibility (e.g., the number of words vs. syllables transcribed) differed across studies, as pointed out by Magrin-Chagnolleau et al. (2002) and Remez et al. (2013). Therefore, perceptual restoration of locally time-reversed speech requires further studies using consistent methodologies, procedures, and measurement.

So far, locally time-reversed speech has been used to discuss: (1) what temporal unit people use in speech perception and perceptual restoration (i.e., which temporal units are accumulated and integrated to understand speech); (2) whether perceptual restoration is language independent or language dependent; and (3) how perceptual restoration is influenced by proficiency in the target language. Magrin-Chagnolleau et al. (2002) suggested that the intelligibility of locally time-reversed speech in French (a syllable-timed language) might differ from that in English (a stress-timed language) (Greenberg & Arai, 2001) with increasing reversed segment length, pointing out a difference in the onset and transition of intelligibility decline – they proposed that this difference might be attributed to differences in language rhythm. Additionally, Kiss et al. (2008) indicated an advantage of native speakers over non-native speakers in perceptual restoration when understanding locally time-reversed German sentences that were semantically coherent (with lexical and sentential context) and semantically incoherent (with lexical context only), although pseudo-homophonic sentences (lacking meaning but with phonologically correct German sounds) were equally unintelligible to both native and non-native speakers. The advantage of native speakers over non-native speakers in perceptual restoration was also confirmed with locally time-reversed English sentences in Ishida et al. (2018), although it is still uncertain how the same individuals perform in their first and second languages (intra-person comparisons which were rarely done in past research). While the perceptual restoration of locally time-reversed speech is presumably sustained by linguistic cues, the mechanism of perceptual restoration in relation to language remains largely to be explored.

As for linguistic factors, locally time-reversed speech has also been discussed in relation to the critical modulation frequency, which is associated with articulatory motions, amplitude envelope, and speech intelligibility. Remez et al. (2013) once analyzed the relationship between reversed segment length and speech intelligibility, converting the duration of the reversed segments (ms) into frequency (Hz), and vice versa, to discuss the critical modulation frequency of speech, as in “between 3 and 8 Hz or 120 and 333 ms.” Here, the duration (ms) was also described as a reflection of linguistic units such as syllables and phonemes that build up a language. However, Ishida et al. (2018) reported that the direct conversion from temporal duration (ms) into frequency (Hz) does not adequately capture the critical modulation frequency of speech, based on two experiments of locally time-reversed speech and modulation-filtered speech in parallel. In these experiments, speech was degraded in six steps (10 ms, 30 ms, 50 ms, 70 ms, 90 ms, and 110 ms for local time reversal degradation, and 32 Hz, 16 Hz, 8 Hz, 4 Hz, 2 Hz, and 1 Hz as low-pass cut-off frequencies for modulation filtering). In general, locally time-reversed English sentences and modulation-filtered English sentences, perceived by native English speakers, showed similar intelligibility decline patterns across the six levels of speech degradation. For example, locally time-reversed English sentences showed half intelligibility when the reversed segment length was increased to 70 ms (= 14 Hz, if converted directly), while modulation-filtered English sentences showed half intelligibility when the modulation frequency components were low-pass filtered with the cut-off frequency of 4 Hz (= 250 ms). Here, the direct conversion of reversed segment length (ms) into frequency (Hz) did not capture the critical modulation frequency of speech in English (with syllables as basic linguistic units that contain consonant clusters), while it remains uncertain whether the same patterns and results would be observed in linguistically different languages such as Japanese (with morae as basic linguistic units consisting of CV and V structures, where vowels typically surround consonants).

Additionally, the perceptual restoration of locally time-reversed and modulation-filtered sentences was influenced by listeners’ language proficiency (Ishida et al., 2018). For example, locally time-reversed English sentences were 97% intelligible for native English speakers and 51% intelligible for non-native English speakers (= L1 Japanese; L2 English with lower intermediate proficiency, as assessed by DIALANG placement test; Alderson, 2006; Lancaster University, ( 2014)) under the least degraded condition, with local time reversals at 10 ms. Similarly, modulation-filtered English sentences were 96% intelligible for native English speakers and 50% intelligible for non-native English speakers under the least degraded condition, with a low-pass cut-off frequency of 32 Hz, where the amplitude contours were relatively preserved. Even with these minimally degraded conditions for both local time reversal and modulation-filtering, there was an obvious gap between native and non-native speakers in perceptual restoration – non-native speakers with lower intermediate proficiency understood only about half of what native speakers understood. At the same time, while non-native speakers are often compared with native speakers of the target language, it is still uncertain how the same individuals understand degraded speech in their first and second languages (intra-person comparisons which are rarely done in past research).

The current study, therefore, explores the effects of language and language proficiency on perceptual restoration by using consistent methodologies, procedures, and measurement from Ishida et al. (2018), while shifting the target language to Japanese. Specifically, this study addresses the following research questions: (1) Does the intelligibility of locally time-reversed speech and modulation-filtered speech differ between Japanese and English when perceived by native speakers? (2) How do native Japanese speakers perceptually restore locally time-reversed speech and modulation-filtered speech in their first and second languages (L1 Japanese vs. L2 English)? (3) Is there any correspondence between reversed segment length (ms) and modulation frequency (Hz)? This study compares the intelligibility of both locally time-reversed and modulation-filtered Japanese sentences, perceived by native Japanese speakers, with those in English reported in Ishida et al. (2018). Notably, the native Japanese speakers in this study are the same individuals who participated in Ishida et al. (2018) as non-native English speakers, allowing for an intra-person comparison of their performance in L1 Japanese and L2 English. The current study investigates perceptual restoration and the intelligibility of locally time-reversed and modulation-filtered sentences in Japanese, which is linguistically different from English and has not been fully investigated in past studies using consistent methodologies.

## Experiment 1: Locally time-reversed speech

Experiment 1 investigates how native Japanese speakers comprehend locally time-reversed Japanese sentences degraded in six steps, following Ishida et al. (2018), to compare perceptual restoration in L1 Japanese versus L1 English (mora-oriented vs. syllable-oriented languages) and perceptual restoration in L1 Japanese versus L2 English (intra-person comparisons).

### Participants

Thirty native Japanese speakers (10 males, 20 females, average age: 34.9 years) participated in this study. These participants were the same individuals who took part in the study by Ishida et al. (2018) as non-native English speakers (native Japanese speakers who spoke English as a foreign language). The sample size of 30 was chosen for consistency based on our previous research on perceptual restoration (Ishida & Arai, 2016; Ishida et al., 2016; Ishida, 2017; Ishida et al., 2018; Ishida, 2021). None of the participants reported any hearing or speech impairments. Prior to participation, all participants submitted consent forms approved by the Institutional Review Board (IRB) of NTT Communication Science Laboratories (H27-010). All data collected were used for analysis, with no exclusions.

### Materials

A total of 12 Japanese sentences was selected from the speech corpus “Onso Balance 1,000 Bun” (NTT Advanced Technology Corporation, 1997), which contains 1,000 sentences designed to be phonemically balanced, and covers all 124 Japanese syllables with 27 Japanese phonemes overall (Appendix A). These sentences, spoken by a professional male narrator, contained an average of 8.83 linguistic elements. The definition of “linguistic elements” in this study was determined considering that Japanese is an agglutinative language where word boundaries are not explicitly indicated by spaces in writing, and there are no definite rules for word segmentation (unlike English, where spaces denote word boundaries in writing) (Kobayashi et al., 2016; Shibatani, 1990). In general, Japanese words can be formed by combining multiple free and/or bound morphemes, and, conversely, a single Japanese word can be segmented into either one or multiple morphemes. For example, the Japanese word “合流した” can be analyzed either as a single Sino-Japanese verb “合流した” (“gooryuushita,” meaning “joined”) or as a word with two linguistic elements: “`合流” (“gooryuu,” a noun meaning “join”) + “した” (“shita,” a verb meaning “did”) (Kobayashi et al., 2016). To define word boundaries, this study used the online Japanese dictionary “Weblio Kokugo Jiten” (GRAS Group Inc., 2024), which houses over 500 Japanese dictionaries. If a target linguistic element (possibly with some morphemes in its structure) is listed as a single word with a meaning in the dictionary, that linguistic element is defined as a word for the current study – i.e., the word boundaries were determined based on the dictionary entry (not based on morphemes). This decision was made to later compare the intelligibility of locally time-reversed sentences in L1 Japanese with those in L1 English (Ishida et al., 2018).

**Fig. 1:**
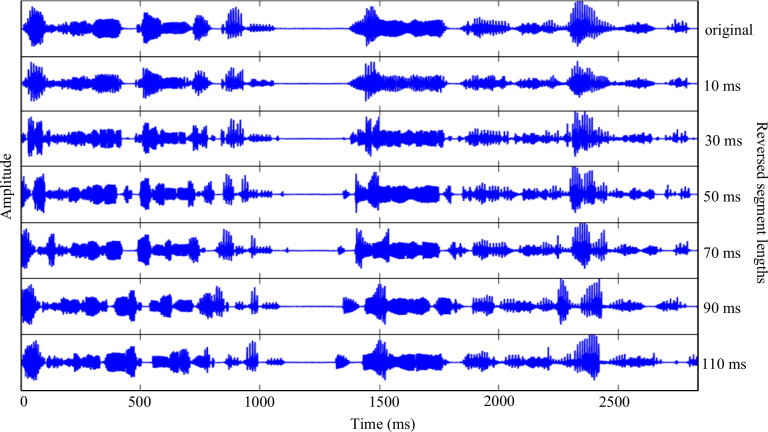
Waveforms of the Japanese sentence “Isshuukan bakari New York wo shuzai shita” (“I stayed in New York for about a week for news coverage”), with time (ms) on the X-axis and amplitude (−0.5 to 0.5) on the Y-axis. The top panel shows the original speech signal. The subsequent panels show locally time-reversed speech signals, where every 10 ms, 30 ms, 50 ms, 70 ms, 90 ms, or 110 ms segment of the signal is reversed in time

The audio files of the selected sentences were stored in WAV format, with a sampling rate of 16,000 Hz and 16-bit resolution (retained from the original corpus recordings). Each speech signal was divided into fixed durations (10 ms, 30 ms, 50 ms, 70 ms, 90 ms, and 110 ms) from the onset of speech, and every segment was reversed along the temporal axis (Fig. 1). The edges of the reversed segments were cross-faded with a tapering length of 5 ms, to prevent additional clicks. The manipulation was conducted using MATLAB, following the procedures established in previous studies of locally time-reversed speech (Ishida et al., 2016; Ishida, 2017, 2021; Ishida et al., 2018).

### Procedure

The experiment was conducted in a sound-shielded room at NTT Communication Science Laboratories, following the procedure of Ishida et al. (2018). The experimental protocol was approved by the IRB of NTT Communication Science Laboratories (H27-010). Participants were seated at a table in front of a computer, wearing headphones (Sony MDR-CD900ST) connected through an audio interface (Roland UA-25 EX). A practice session was conducted in which participants listened to and transcribed two locally time-reversed sentences, not used in the main experiment, to familiarize them with the task and check the audio level and experimental procedure.

In the experiment, each participant listened to a set of 12 Japanese sentences in a fixed order and transcribed what they heard using pen and paper, writing next to the corresponding trial number. Participants were instructed to write their answers as they would in daily life, using any combination of Hiragana, Katakana, Kanji, or Roman alphabets – the four character types used in Japanese writing. They were encouraged to use the characters they felt best represented what they heard. Each sentence was presented after a 20-s silent interval, and participants were asked to write their responses as quickly as possible. After listening to the set of 12 sentences, they listened to the entire set again to check the legibility of their transcriptions and make any necessary corrections. For the first listening, they used a blue pen, and for the second, a red pen. The set of 12 sentences consisted of six subsets, each containing two sentences. Each subset was assigned to one of six reversed segment lengths (10, 30, 50, 70, 90, or 110 ms). Participants were randomly divided into six groups, and a Latin square design was used to counterbalance the subsets of sentences across the six reversed segment lengths. The entire experiment lasted approximately 30 min.

### Results

The intelligibility of locally time-reversed sentences in L1 Japanese was assessed by the proportion of correctly transcribed words relative to the total number of words per sentence for each participant. A correct transcription was defined as a match between the spoken sounds and the transcribed words (Kawagoe, 2007). Transcriptions were considered correct if they could be read as the intended spoken sounds, even if the choice of characters was unusual or non-standard. For example, the transcription of “isshuukan” (meaning “1 week”) was accepted in various forms such as 一週間 (Chinese numeral + Kanji), 1週間 (Arabic numeral + Kanji), 一週かん (Chinese numeral + Kanji + Hiragana), or いっしゅうかん (all in Hiragana), as long as it could be read as “isshuukan.” Similarly, “nyuuyooku” (meaning “New York”) was considered correct when transcribed as ニューヨーク (all in Katakana), New York (Roman alphabet), or NY (abbreviation in Roman alphabet). For the sounds “kyuumei,” acceptable transcriptions included 究明 (meaning “investigation” in Kanji), きゅう明 (Hiragana + Kanji), きゅうめい (all in Hiragana), and 救命 (“lifesaving” in Kanji), since all forms could be read as “kyuumei.” Homophones with different Kanji were accepted as long as they were phonetically identical and meaningful; the limitation of this approach is also discussed further in the paper. Since Japanese writing uses four different scripts (Hiragana, Katakana, Kanji, and the alphabet), participants were allowed to transcribe in their natural style, reflecting their perception and perceptual restoration.

**Fig. 2:**
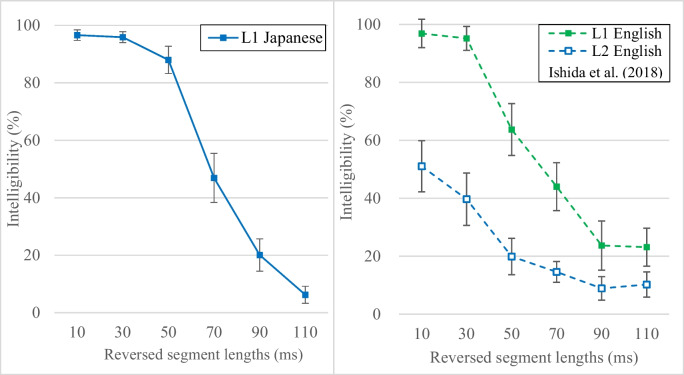
Intelligibility of locally time-reversed sentences in L1 Japanese (**left panel**), compared with that in L1 English and L2 English reported in Ishida et al. (2018) (**right panel**). The L1 Japanese and L2 English data were obtained from the same individuals—native Japanese speakers who spoke English as a second language. The L1 English data were collected from 30 native English speakers at Stony Brook University (25 females, five males; average age: 19.9 years). Both the L1 English and L2 English participants in Ishida et al. (2018) listened to 18 English sentences from *Listen, Read, and Write: Sentences for Sight Word Dictation* (Wickham, 2013), which were locally time reversed

The results indicated that locally time-reversed sentences in L1 Japanese were 97%, 96%, 88%, 47%, 20%, and 6% intelligible at reversed segment lengths of 10, 30, 50, 70, 90, and 110 ms, respectively (Fig. 2). This pattern aligns with the general trends of intelligibility decline with increasing reversed segment length reported in L1 English (Ishida et al., 2018), where intelligibility was 97%, 95%, 64%, 44%, 24%, and 23% for the same reversed segment lengths under corresponding experimental conditions. While locally time-reversed sentences in L1 Japanese retained much higher intelligibility at the 50-ms reversed segment length and lower intelligibility at 100 ms as compared with L1 English, intelligibility at other segment lengths was nearly equivalent across both languages.

To examine whether the intelligibility patterns in listeners’ first language were consistent between L1 Japanese and L1 English, an ANOVA was performed with language (L1 Japanese vs. L1 English) as a between-subject factor and reversed segment length (10 ms, 30 ms, 50 ms, 70 ms, 90 ms, and 110 ms) as a within-subject factor, using the L1 English data from Ishida et al. (2018). The results indicated a significant overall difference in intelligibility between L1 Japanese and L1 English, *F* (1, 58) = 0.47, *p* = 0.0498, ${\eta }_{p}^{2}$= 0.01. Intelligibility also deteriorated significantly with increasing reversed segment length, *F* (3.11, 180.43) = 267.93, *p* < 0.001, ${\eta }_{p}^{2}$= 0.82 (Greenhouse-Geisser correction; Greenhouse & Geisser, 1959). Additionally, a significant interaction between language and reversed segment length was observed, *F* (3.11, 180.43) = 9.05, *p* < 0.001, ${\eta }_{p}^{2 }$= 0.14, indicating that the rate of intelligibility declines with increasing reversed segment length differed between L1 Japanese and L1 English. Follow-up independent *t*-tests (Table 1) also indicated that intelligibility of locally time-reversed sentences in L1 Japanese and L1 English was similar across most segment lengths, with significant differences observed at 50 ms and 110 ms.

**Table 1:** Results of Follow-up t-tests Comparing Intelligibility of Locally Time-Reversed Sentences in L1 Japanese and L1 English Across Different Segment Lengths

Reversed Segment Length	Japanese		English		*t*-value (*df*)	*p*-value	95% CI	Cohen's *d*
	*M*	*SD*	*M*	*SD*				
10 ms	96.61	5.09	96.87	13.67	–0.10 (58)	0.92	[–5.59, 5.07]	0.03
30 ms	95.89	5.30	95.18	11.55	0.30 (58)	0.76	[–3.94, 5.35]	0.08
50 ms	87.98	13.26	63.72	24.94	**4.71 (44.18)**	< 0.001*	**[13.87, 34.65]**	**1.21**
70 ms	46.91	23.86	44.01	23.10	0.48 (58)	0.64	[–9.24, 15.03]	0.12
90 ms	20.09	15.70	23.69	23.71	–0.69 (58)	0.49	[–13.99, 6.79]	0.18
110 ms	6.25	8.30	23.12	18.26	**–4.61 (40.49)**	< 0.001*	**[**–**24.27,** –**9.48]**	**1.19**

*Note.* The asterisks (*) indicate a significant difference after applying the Bonferroni correction (α = 0.0083) for multiple comparisons

At the same time, comparing perceptual restoration of locally time-reversed sentences in listeners’ first language (L1 Japanese) and second language (L2 English, with lower intermediate proficiency; data from Ishida et al., 2018), a clear disadvantage was observed in the second language: intelligibility in L2 English was 51%, 41%, 20%, 15%, 9%, and 10% for the same reversed segment lengths under corresponding experimental conditions. While the intelligibility in the listeners’ first language was 90% with the lightest degradation at 10 ms, perceptual restoration in listeners’ second language began at about half the intelligibility. While non-native speakers are often compared with native speakers of the target language as a baseline to highlight the native speaker advantage (e.g., L1 vs. L2 English as in Ishida et al., 2018), this study also illustrated the advantage of listeners’ first language over their second language in perceptual restoration (L1 Japanese vs. L2 English) within the same individuals.

Overall, the intelligibility of locally time-reversed sentences in L1 Japanese was significantly different from that in L1 English, particularly at 50 ms and 110 ms. However, the general trend of intelligibility decline with increasing reversed segment length was similar across both languages. Additionally, the advantage of native language in perceptual restoration was confirmed within the same individuals when comparing their performance in their first language (L1 Japanese) as a baseline with that in their second language (L2 English, with lower intermediate proficiency), as was also observed in the comparison between native and non-native English speakers reported in Ishida et al. (2018).

## Experiment 2: Modulation-filtered speech

Experiment 2 investigates how native Japanese speakers comprehend modulation-filtered speech in Japanese, degraded in six steps, following Ishida et al. (2018). The Japanese spoken sentences were gradually degraded by low-pass filtering the modulation frequency components that determine the temporal configuration of the speech signal. This experiment compares perceptual restoration of modulation-filtered speech in L1 Japanese versus L1 English (mora-oriented vs. syllable-oriented languages) and perceptual restoration in L1 Japanese vs. L2 English (intra-person comparisons), with the English data from Ishida et al. (2018). Additionally, the intelligibility of modulation-filtered speech will later be compared with that of locally time-reversed speech from Experiment 1 to discuss the relationship between reversed segment length and modulation frequency of speech.

### Participants

The participants were the same as those in Experiment 1. Prior to participation, all participants submitted consent forms approved by the IRB of NTT Communication Science Laboratories (H27-010). All data collected were used for analysis, with no exclusions.

### Materials

A total of 12 Japanese sentences spoken by a professional male narrator were newly selected from the speech corpus “Onso Balance 1,000 Bun” (phonemically balanced 1,000 sentences) by NTT Advanced Technology Corporation (1997) (Appendix B). The audio files were stored in WAV format with a sampling rate of 16,000 Hz and 16-bit resolution (retained from the original corpus recordings). The average number of Japanese linguistic elements per sentence (used for the evaluation of intelligibility) was 9.75. The linguistic elements (i.e., word boundaries) were determined using “Weblio Kokugo Jiten” (GRAS Group Inc., 2024), as in Experiment 1.

**Fig. 3:**
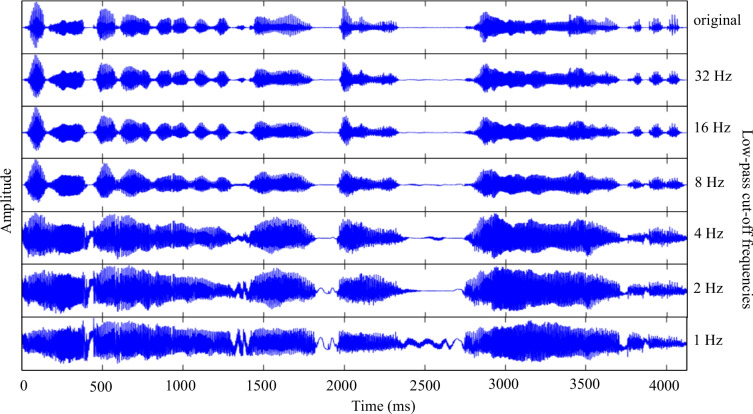
The speech signals of the Japanese sentence “Hajimete Louvre bijutsukan e itta nowa 14nen mae no koto da” (It was 14 years ago that I went to the Louvre Museum for the first time), with time (ms) on the X-axis and amplitude (−1 to 1) on the Y-axis. The top panel shows the original speech signal. The subsequent panels show the speech signals of modulation-filtered speech, where the modulation frequency components are low-pass filtered at cut-off frequencies of 32, 16, 8, 4, 2, and 1 Hz

To modify the temporal envelope of the speech signal, the current study followed the procedures of Ishida et al. (2018), which are based on Greenberg et al. (1998). First, the speech signal, with a maximum frequency of 6,000 Hz, was divided into 1/3-octave bands, resulting in 14 frequency bands: 13 1/3-octave-wide channels and one remaining channel. These 14 frequency bands cover the following ranges: 0–298 Hz, 298–375 Hz, 375–473 Hz, 473–595 Hz, 595–750 Hz, 750–945 Hz, 945–1,191 Hz, 1,191–1,500 Hz, 1,500–1,890 Hz, 1,890–2,381 Hz, 2,381–3,000 Hz, 3,000–3,780 Hz, 3,780–4,762 Hz, and 4,762–6,000 Hz. The lowest frequency band (0–298 Hz) represents the remaining frequencies, which were low-pass filtered (*Note*: The critical band analysis was approximated using 1/3-octave band analysis, as 1/3-octave bands are thought to closely match the width of human auditory critical bands, particularly in mid-to-high frequencies (Moore, 2012)). The FIR (finite impulse response) filter was designed using the Kaiser window (Kaiser, 1966; Oppenheim et al., 1999) with a transition length of 100 Hz and a peak approximation error of *δ* = 0.001, and the filter slopes exceeded 100 dB/oct. Next, the amplitude envelope of each band signal was computed using the Hilbert Transform (Rabiner & Gold, 1975). The computed envelope was then low-pass filtered with cut-off frequencies of 32, 16, 8, 4, 2, or 1 Hz. These cut-off frequencies were chosen based on Ishida et al. (2018), which showed a gradual intelligibility decline starting from 96% at the highest cut-off to 17% at the lowest, using six steps. This manipulation was carried out with another FIR filter, also designed with the Kaiser window, featuring a transition length of 1 Hz and a peak approximation error of *δ* = 0.1. The modified band signals were combined to form the amplitude-modulated speech signal (Fig. 3). The sound level was normalized based on the RMS (root mean square) value, and all manipulations were performed using MATLAB.

### Procedure

The experimental procedure was based on Ishida et al. (2018). The devices and environment were the same as in Experiment 1. The experimental protocol was approved by the IRB of NTT Communication Science Laboratories (H27-010).

Participants were randomly assigned to one of six groups in a Latin square design. They listened to a set of 12 sentences and transcribed what they heard next to the corresponding trial number on the answer sheet using a pen. There were six subsets of two sentences, with each subset assigned a different low-pass cut-off frequency of 32 Hz, 16 Hz, 8 Hz, 4 Hz, 2 Hz, or 1 Hz. The six groups of participants counterbalanced these six subsets across the six low-pass cut-off frequencies. For transcription, participants were instructed to write their responses as they would naturally in daily life, using any combination of Japanese characters (Hiragana, Katakana, Kanji) and the Roman alphabet. They were asked to write their answers as quickly as possible, as the next sentence was played after a 20-s silence interval. After listening to all 12 sentences, participants listened to the same set again to check and correct their transcription for legibility, using a different color pen. The first transcription was done in blue, and the second in red. The entire experiment took approximately 30 min.

### Results

The intelligibility of modulation-filtered Japanese sentences was assessed by calculating the proportion of correctly transcribed words relative to the total number of words, following the same procedure as Experiment 1. Evaluations were based on the correspondence between the spoken stimuli and written transcriptions (Kawagoe, 2007). Transcriptions were accepted as long as they conveyed the spoken message, even if the choice of characters was unusual, non-standard, or both. For example, the word “oosakaben” (meaning *Osaka dialect*) was accepted as 大阪弁 (all in Kanji), 大阪べん (Kanji + Hiragana), 大さかべん (Kanji + Hiragana), and おおさかべん (all in Hiragana), as all versions represent the same speech sounds. Similarly, “shichuu” (meaning “stew”) was accepted as シチュー or シチュウ (both in Katakana), reflecting variations in Katakana transcription for elongated sounds. The word “Hajimete” (meaning “for the first time”) was accepted in the forms as初めて (Kanji+ Hiragana), はじめて (all in Hiragana), and 始めて (Kanji + Hiragana). Kanji transcriptions were accepted if they were phonetically identical to the spoken words, regardless of whether the Kanji has the same or different meanings – this limitation is discussed further in the *Discussion* section. Given that Japanese writing combines four different character sets (Hiragana, Katakana, Kanji, and Roman alphabet), participants were allowed to use any form they preferred to represent what they perceived and restored perceptually.

**Fig. 4:**
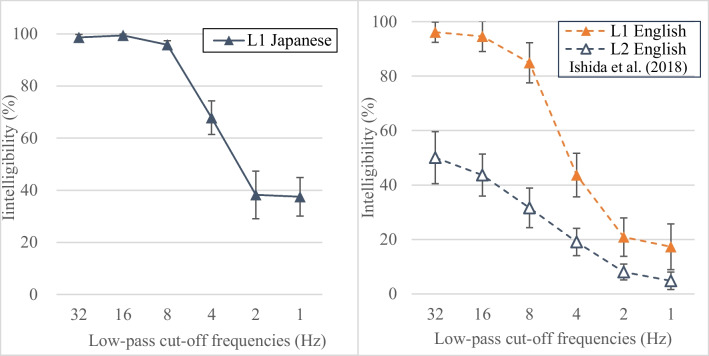
Intelligibility of modulation-filtered speech in L1 Japanese (**left panel**), compared with that in L1 English and L2 English data reported in Ishida et al. (2018) (**right panel**). The *L1 Japanese* and *L2 English* data were obtained from the same individuals: native Japanese speakers who spoke English as a second language. The L1 English data were collected from 30 native English speakers at Stony Brook University (25 females, five males; average age: 19.9 years). Both L1 English and L2 English participants in Ishida et al. (2018) listened to 18 English sentences from *Listen, Read, and Write: Sentences for Sight Word Dictation* (Wickham, 2013), in which the modulation frequency components of speech were low-pass filtered

The results showed that modulation-filtered sentences in L1 Japanese were 99%, 99%, 96%, 68%, 38%, and 38% intelligible at low-pass cut-off frequencies of 32, 16, 8, 4, 2, and 1 Hz, respectively (Fig. 4). This pattern aligns with the general trends of intelligibility decline with decreasing low-pass cut-off frequencies observed in L1 English (Ishida et al., 2018), where intelligibility was 96%, 95%, 85%, 44%, 21%, and 17%, respectively, under corresponding experimental conditions. What was particularly remarkable, however, is that the intelligibility of modulation-filtered sentences in L1 Japanese was generally higher across all conditions compared to L1 English. This suggests that L1 Japanese may be more tolerant of modulation filtering, potentially due to its language structure, which is characterized by alternating vowels and consonants in CV and V patterns (which is discussed later).

To examine whether the intelligibility patterns differed between L1 Japanese and L1 English, an ANOVA was conducted with language (Japanese vs. English) as a between-subject factor and low-pass cut-off frequencies (32, 16, 8, 4, 2, and 1 Hz) as a within-subject factor, using the L1 English data from Ishida et al. (2018). The results indicated a significant overall difference in intelligibility between L1 Japanese and L1 English, *F* (1, 58) = 69.61, *p* < 0.001, ${\eta }_{p}^{2}$= 0.55. Intelligibility also significantly deteriorated with decreasing low-pass cut-off frequencies, *F* (2.83, 163.87) = 205.22, *p* < 0.001, ${\eta }_{p}^{2}$= 0.78 (Greenhouse-Geisser correction; Greenhouse & Geisser, 1959). A significant interaction was observed between language and low-pass cut-off frequencies, *F* (2.83, 163.87) = 3.55, *p* = 0.018, ${\eta }_{p}^{2}$ = 0.06, indicating that the rate of intelligibility declines with decreasing low-pass cut-off frequencies differed between L1 Japanese and L1 English. Follow-up independent *t*-tests (Table 2) revealed that the intelligibility of modulation-filtered sentences in L1 Japanese differed significantly from that in L1 English, particularly at the low-pass cut-off frequencies of 8 Hz, 4 Hz, 2 Hz, and 1 Hz, highlighting a difference at lower modulation frequencies.

**Table 2:** Results of Follow-up t-tests Comparing the Intelligibility of Modulation-filtered Sentences in L1 Japanese and L1 English Across Different Low-pass Cut-off Frequencies

Low-pass Cut-off Frequency	Japanese		English		*t*-value (*df*)	*p*-value	95% CI	Cohen's *d*
	*M*	*SD*	*M*	*SD*				
32 Hz	98.65	3.14	96.17	10.37	1.25 (34.28)	0.22	[–1.54, 6.50]	0.32
16 Hz	99.42	1.52	94.62	15.54	1.68 (29.55)	0.103	[–1.03, 10.62]	0.43
8 Hz	95.83	4.41	84.91	20.65	**2.83 (31.64)**	0.008*	**[3.07, 18.78]**	**0.73**
4 Hz	67.83	17.97	43.65	22.44	**4.61 (58)**	< 0.001*	**[13.68, 34.69]**	**1.19**
2 Hz	38.26	25.52	20.86	19.69	**2.96 (58)**	0.005*	**[5.61, 29.17]**	**0.76**
1 Hz	37.53	20.71	17.3	23.53	**3.54 (58)**	0.001*	**[8.78, 31.69]**	**0.91**

*Note.* The asterisks (*) indicate a significant difference after applying the Bonferroni correction (α = 0.0083) for multiple comparisons

When comparing the perceptual restoration of modulation-filtered sentences by the same listeners in their first language (L1 Japanese) and second language (L2 English, with lower intermediate proficiency; data from Ishida et al., 2018), a clear disadvantage in the second language was observed. The intelligibility of L2 English sentences was 50%, 44%, 32%, 19%, 8%, and 5% under corresponding experimental conditions. Here, perceptual restoration in listeners’ second language began at about half the intelligibility of that in their first language. This study therefore highlights the advantage of listeners’ first language over their second language in perceptual restoration (L1 Japanese vs. L2 English), using L1 performance as a baseline, in addition to the native speaker advantage over non-native speakers reported in Ishida et al. (2018).

Additionally, when comparing the intelligibility of modulation-filtered sentences from Experiment 2 (99%, 99%, 96%, 68%, 38%, and 38% at low-pass cut-off frequencies of 32, 16, 8, 4, 2, and 1 Hz) with that of locally time-reversed sentences from Experiment 1 (97%, 96%, 88%, 47%, 20%, and 6%), no direct correspondence between the modulation frequency and reversed segment length was found. While both experiments started with over 95% intelligibility at the first two lightest degradation levels (low-pass cut-off frequencies of 32 Hz and 16 Hz, and reversed segment lengths of 10 ms and 30 ms), the direct conversion between frequencies (Hz) and durations (ms) does not show a corresponding relationship – for instance, 32 Hz corresponds to 31 ms if converted directly, 16 Hz corresponds to 63 ms, 10 ms corresponds to 100 Hz, and 30 ms corresponds to 33 Hz. As pointed out in Ishida et al. (2018), local time reversal and modulation filtering are different types of speech degradation, and thus, the direct conversion from duration (ms) to frequency (Hz) does not accurately describe the critical modulation frequency of speech.

Overall, the intelligibility of modulation-filtered speech in L1 Japanese was significantly different from that in L1 English, with L1 Japanese retaining higher intelligibility across all low-pass cut-off frequencies. While intelligibility decreased with decreasing low-pass cut-off frequencies in both languages, L1 Japanese consistently demonstrated significantly higher intelligibility, particularly 8 Hz, 4 Hz, 2 Hz, and 1 Hz. When the amplitude envelope of speech signals was smeared using modulation filtering, speech was significantly more intelligible in L1 Japanese than in L1 English. Additionally, the advantage of the native language was confirmed within the same individuals when comparing perceptual restoration of modulation-filtered speech in their first language (L1 Japanese) with that in their second language (L2 English, with lower intermediate proficiency). At the same time, there was no correspondence between modulation frequencies and reversed segment length when comparing the intelligibility of Experiment 2 with Experiment 1.

### Discussion

The current study explored perceptual restoration by examining how native Japanese speakers perceive locally time-reversed and modulation-filtered Japanese sentences, compared to native and non-native English speakers reported in Ishida et al. (2018). Specifically, this study investigated (1) the effects of language structure (mora-based Japanese vs. syllable-based English) on perceptual restoration, (2) the role of language proficiency (L1 Japanese vs. L2 English within the same individuals), and (3) the relationship between reversed segment length (ms) and modulation frequency (Hz) through two parallel experiments. This discussion session focuses on these points in that order.

### Language structure and perceptual restoration

Regarding (1) the effects of language structure on perceptual restoration, the current study revealed significant differences between L1 Japanese and L1 English. While intelligibility declined gradually with increasing degradation levels across six steps in both Experiment 1 and Experiment 2, the patterns of decline differed significantly between Japanese and English.

For example, the intelligibility of locally time-reversed speech in L1 Japanese was generally equal to or higher than that in L1 English up to a reversed segment length of 70 ms (where intelligibility dropped by half in both languages, consistent with past studies). Multiple comparisons also showed significant differences at 50 ms (88% vs. 64% for L1 Japanese vs. L1 English) and 110 ms (6% vs. 23 %). Here, the higher intelligibility of L1 Japanese, especially at shorter reversed segment lengths, might be attributable to the basic linguistic units of Japanese (CV and V), where a consonant is always followed by a vowel in a sentence and a word always ends with a vowel; in other words, vowels surround a consonant most of the time. In fact, vowels are highly tolerant of temporal degradation. Pellegrino et al. (2010) reported that vowels in CVC pseudowords in French retained high intelligibility (89%) even when globally inverted (i.e., played backwards), while the intelligibility of consonants varied depending on their type: liquids (96%), unvoiced fricatives (93%), voiced fricatives (92%), nasals (90%), rhotics (67%), voiced stops (62%), schwa (25%), nasal vowels (17%), and unvoiced stops (9%). When vowels appear in CV or V sequences in Japanese, alternating with consonants, their intelligibility can reasonably be assumed to remain high even when temporally inverted. L1 Japanese listeners may be particularly familiar with these sequences and their associated amplitude envelope, while drastic temporal inversions at longer reversed segment lengths would more severely alter the temporal configuration and CV sequence of the speech signal, leading to lower intelligibility due to unsuccessful perceptual restoration.

Additionally, the intelligibility of modulation-filtered sentences in L1 Japanese was higher than in L1 English across all degradation levels. While intelligibility declined in both languages as the low-pass cut-off frequency decreased, L1 Japanese consistently outperformed L1 English. Multiple comparisons also revealed that L1 Japanese was significantly more intelligible than L1 English at cut-off frequencies of 8 Hz (96% vs. 85% for L1 Japanese vs. L1 English), 4 Hz (68% vs. 44%), 2 Hz (38% vs. 21%), and 1 Hz (38% vs. 17%). Here, L1 Japanese intelligibility remained more than twice that of L1 English at 1 Hz (the most severe temporal degradation). This may again be attributable to the regular occurrence of vowels and CV and V sequences in Japanese sentences. In fact, Drullman et al. (1994) reported that vowels were highly tolerant of modulation filtering compared to consonants. In their study, speech signals of CVC and VCV meaningless syllables in Dutch were divided into 1/4-octave bands (“just smaller than the ear’s critical bandwidth”) and each band was low-pass filtered at cut-off frequencies of 16 Hz, 8 Hz, 4 Hz, 2 Hz, and 0 Hz, then combined. The target for identification in CVC was the initial consonant, vowel, and final consonant, while in VCV it was the consonant. Their results showed that vowels remained 56% intelligible at a low-pass cut-off frequency of 0 Hz (the most severe temporal smearing with no modulation), which was double the average intelligibility of consonants. When a language has CV and V as its basic linguistic units, with vowels surrounding consonants, vowels would provide both acoustic and contextual cues for perceptual restoration.

Comparing the basic linguistic unit of Japanese (morae) and English (syllables), there is a substantial gap between the two languages (Table 3). While Japanese has CV and V as the basic linguistic units of a mora, with C and CCV as “special morae,” English has a complex syllable structure with a variety of consonant clusters: CV, V, VC, VCC, VCCC, CVC, CVCC, CVCCC, CVCCCC, CCV, CCVC, CCVCC, CCVCCC, CCCV, CCCVC, CCCVCC, CCCVCCC, and CVCCCC (Bergman et al., 2007; Celce-Murcia et al., 2010; Kono, 2004). Just by looking at the basic linguistic units, Japanese has only two basic mora structures (or four when including special morae), while English has up to 18 syllable structures. This gap in basic linguistic units between the two languages could have a substantial effect on perceptual restoration. The regular occurrence of vowels in CV and V morae in Japanese would likely help sustain the intelligibility of acoustically degraded sentences, such as locally time-reversed speech and modulation-filtered speech. Vowels likely provide essential cues for perceptual restoration.

**Table 3:** Overview of the Japanese Mora Structure vs. the English Syllable Structure (cf. Kono, 2004; Bergman et al., 2007; Celce-Murcia et al., 2010; Ishida, 2021)

**Japanese**			
Basic mora	Example word	IPA	Meaning
CV	手	/te/	hand
V	尾	/o/	tail
Special mora	Example word	IPA	Meaning
C	葉っぱ	/Q/ in /haQpa/ (3morae)	leaf
	先生	/N/ in /seNsei/ (4 morae)	teacher
CCV	旅行	/rjo/ in /rjokou/ (3 morae)	travel
**English**			
Syllables	Example word	IPA	
V	a, eye, I	/ə/, /aɪ/	
VC	at	/æt/	
VCC	and	/ænd/	
VCCC	inks	/ɪŋks/	
CV	go	/ɡoʊ/	
CVC	pen	/pɛn/	
CVCC	jump	/dʒʌmp/	
CVCCC	camps	/kæmps/	
CVCCCC	texts	/tɛksts/	
CCV	play	/pleɪ/	
CCVC	flip	/flɪp/	
CCVCC	plump	/plʌmp/	
CCVCCC	stamped	/stæmpt/	
CCVCCCC	twelfths	/twɛlfθs/	
CCCV	straw	/strɑː/	
CCCVC	splash	/splæʃ/	
CCCVCC	straps	/stræps/	
CCCVCCC	scrimps	/skrɪmps/	

### Language proficiency and perceptual restoration

With regard to (2), the effect of language proficiency on perceptual restoration, the current study revealed a substantial gap between listeners’ first language (L1 Japanese) and second language (L2 English with lower intermediate proficiency) within the same individuals. Even under the lightest speech degradation, with a reversed segment length of 10 ms and a low-pass cut-off frequency of 32 Hz, perceptual restoration was challenging in the second language. While the intelligibility of locally time-reversed and modulation-filtered speech in L1 Japanese was 97% and 99%, respectively, it was 51% and 50% in L2 English (with lower intermediate proficiency). The same individuals comprehended degraded speech almost perfectly in their first language, yet their understanding dropped by half in their second language. Here, their auditory processing ability at the bottom-up level should be the same for both their first and their second languages, but perceptual restoration differed significantly – perceptual restoration was strongly influenced by listeners’ language proficiency.

In fact, perceptual restoration involves the integration of both bottom-up acoustic/phonemic cues and top-down contextual/linguistic cues (Liberman et al., 1967), and this process is highly regulated by listeners’ language proficiency and familiarity with the topic (Bond, 1999; Brown & Kondo-Brown, 2006; Cherry & Wiley, 1967; Ishida & Arai, 2016; Nation, 2001, 2006; Voss, 1984; Warren & Obusek, 1971; Warren & Sherman, 1974). If listeners are familiar with the sound rules of the target language (e.g., phonemes, phonology, phonotactics, coarticulation, intonation, and rhythm), they would have some expectations of upcoming sounds based on coarticulation. In fact, Kashino et al. (1992) suggested that Dutch listeners outperformed Japanese listeners in perceptually restoring a stop consonant in VC (extracted from VCV) and VC_1_-C_2_V nonsense sequences (where the pre- and post-closure portions of two different stop consonants are combined), uttered by a native Japanese speaker, with the post-closure portion replaced by the noise of 0 ms, 10 ms, 30 ms, 50 ms, and 70 ms. Here, Dutch listeners had VC sequences as their basic linguistic unit in their native language, while Japanese do not have it, leading the Dutch listeners to be more successful in perceptual restoration based on the coarticulatory cues. Acoustic information based on listeners’ native language and associated coarticulation provide cues for restoration.

Additionally, if listeners are familiar with the formation of words and sentences, as well as how the target language is used in different contexts (e.g., morphology, syntax, semantics, pragmatics, background knowledge, familiarity with the topic and vocabulary), they would be able to speculate about what is being discussed in the given speech. In fact, Warren (1970) first reported Gary Sherman’s unpublished study, where the missing sound (*) in “(*)ite” within a sentence, replaced by noise, was perceptually restored as “bite” or “fight” when a preceding or following part of the sentence described a snarling dog, although the word could have been “kite,” “light,” or “white.” Warren and Obusek (1971) also reported that the missing sound (*) in “There was time to (*)ave” was perceptually restored as “wave” when the sentence described a friend departing, although it could have been “save,” “rave,” or “shave.” While these examples illustrate how contextual information affects perceptual restoration by native speakers of English, listeners with limited vocabulary and familiarity with the target language might not show the same results, as they lack sufficient top-down linguistic expectations. Listeners’ expectations, based on their linguistic knowledge and familiarity with the topic, greatly affect perceptual restoration.

At the same time, perceptual restoration is not always successful, even when listeners are familiar with the target language and the topic (Bond, 1999; Brown & Kondo-Brown, 2006; Kashino & Craig, 1994; Voss, 1984). Listeners can misperceive speech and experience “slips of the ear” even when they are attentive to the speaker. While misperception often occurs when a speaker does not pronounce the target word clearly or omits some phonemes in articulation (Bond, 1999; Voss, 1984), there is no simple cause-and-effect relationship in “slips of the ear” (i.e., unsuccessful perceptual restoration). For example, native English speakers perceived “There’s some ice tea made” as “There’s a nice teammate” and “I have to eat” as “I have eighty-two” (Bond, 1999). Additionally, in a second language, native German speakers perceived the English sentence “we don’t want people to think” as “we don’t want paper to think” and “(we are) honored to be sure” as “on a TV show” (Voss, 1984). Furthermore, native Japanese speakers perceived the English word “hardware” in a sentence as “haraware” and “stairway” as “stayaway,” respectively, inserting vowels into consonant clusters (Kashino & Craig, 1994). Since English involves connected speech, where phonemes and vowels are combined, dropped, and pronounced together (e.g., “J'eat yet?” for “Did you eat yet?”; Brown, 2006; Brown & Hilferty, 1982, 1986, 1995), listeners must perceptually restore parts that were not clearly articulated (Brown & Kondo-Brown, 2006; Dalby, 1986; Johnson, 2004). This process requires not only linguistic knowledge but also pragmatic knowledge of how language sounds and is used in real life – giving native speakers an advantage in perceptual restoration compared to non-native speakers. When connected speech is degraded acoustically, as in locally time-reversed speech and modulation-filtered speech, perceptual restoration becomes even more difficult for non-native speakers.

### Reversed segment length and modulation frequency

For (3), the relationship between reversed segment length and modulation frequency (Hz), there was no direct correspondence between the duration of the reversed segment (ms) and modulation frequency of speech (Hz). For example, the intelligibility of locally time-reversed Japanese sentences (97%) and modulation-filtered Japanese sentences (99%) was almost equivalent at the lightest degradation levels – 10 ms and 32 Hz, respectively. However, directly converting the reversed segment duration into frequency did not capture the modulation frequency of speech: 10 ms corresponds to 100 Hz, while 32 Hz corresponds to 31 ms. While both locally time-reversed and modulation-filtered speech in L1 Japanese showed a gradual decline in intelligibility across six levels of degradation (as also observed in L1 English), there was no direct correspondence between reversed segment length and modulation frequency.

At the same time, the intelligibility decline patterns of locally time-reversed speech and modulation-filtered speech in L1 Japanese differed from those in L1 English. Specifically, locally time-reversed speech in L1 Japanese started with higher intelligibility at shorter reversed segment lengths and ended with lower intelligibility at longer reversed segment lengths than in L1 English, whereas modulation-filtered speech in L1 Japanese remained consistently more intelligible across all six degradation levels. One possible explanation for why L1 Japanese exhibited higher intelligibility at shorter segment lengths but lower intelligibility at longer reversed segment lengths compared to L1 English under local time reversal is that L1 Japanese listeners are familiar with CV sequences and rely on their expectation of the temporal sequence (i.e., phonotactic rules) for perceptual restoration (Ishida, 2021; Kashino, 1990; Kashino et al., 1992). This expectation facilitates perception, particularly at shorter segment lengths, whereas at longer segment lengths, the CV structure and the temporal configuration of the acoustic signal are drastically altered. When local time reversal is applied at longer segment lengths, the temporal constituents of the speech signal become more widely dispersed in time, shifting the acoustic components forward or backward. On the other hand, modulation-filtered speech in Japanese was consistently more intelligible than in English, likely due to the characteristics of acoustic degradation and the structural properties of the Japanese language. Specifically, modulation filtering as a form of acoustic degradation does not alter the temporal position of speech. Unlike locally time-reversed speech, modulation filtering simply reduces the temporal components of speech (i.e., modulation frequency), while preserving articulatory motions from the onset to the offset of speech. The regular occurrence of vowels in Japanese CV structure would provide consistent temporal and perceptual cues as degradation levels increase with modulation filtering.

Overall, modulation-filtered speech in the current study showed a gradual intelligibility decline across six steps. Additionally, the results revealed the 3- to 8-Hz range as critical for speech perception – with 4 Hz being the most critical (68% for L1 Japanese, 44% for L1 English), consistent with previous studies (Arai & Greenberg, 1998; Drullman et al., 1994; Greenberg & Arai, 2001, 2004; Greenberg et al., 2006; Ishida et al., 2018; Kanedera et al., 1998). However, the critical modulation frequency cannot simply be inferred by solely converting the length of the critical reversed segment of locally time-reversed speech (ms) into frequency (Hz). Since local time reversal and modulation-filtered speech represent different types of acoustic degradation – differing in whether the natural articulatory motion in its temporal order is preserved or altered – their effects impact Japanese and English differently in relation to the structure of basic linguistic unit and listeners’ language proficiency.

### Limitations: Perceptual restoration research with japanese sentences

One possible limitation that future research on speech perception should recognize when using Japanese sentences is that the Japanese language contains many homophones with identical accent patterns. For example, “seikaku”/seikaku/can be understood as “正確” (accurate) or “性格” (personality). Also, “kouen”/kouen/can be understood as “公園” (park), “講演” (lecture), “公演” (performance), or “後援” (sponsorship). When examining perceptual restoration of degraded speech at the sentence level using transcription, words can serve as ideal units of measurement to assess which sounds listeners grouped together to recognize words within a given context. However, allowing listeners to transcribe words freely, as they naturally would in daily life, means Japanese listeners would likely write their answers by combining Hiragana, Katakana, Kanji, and the Roman alphabet. In this natural writing style, two types of transcription errors might occur: (1) listeners accurately perceive the sound and meaning of the target word (i.e., they correctly capture word boundaries and context) but make transcription errors due to typos or unusual letter choices; or (2) listeners capture the sound of the target word (with the correct number of morae) but misunderstand its meaning in context, leading to transcription errors. In both cases, listeners recognize the specific number of sounds as a single word, yet it remains challenging to determine whether the mistranscription resulted from typographical errors or misinterpretation. One possible countermeasure is to have listeners listen to the stimuli set again, and check and correct their answers themselves if needed. Another approach is to ask listeners to transcribe using a single type of script (e.g., Hiragana only or Katakana only), though this may introduce an additional cognitive load, as adult native Japanese speakers typically combine scripts to visually and functionally indicate word boundaries and meanings. These limitations persist as long as transcription is used as a measurement of perceptual accuracy and perceptual restoration for Japanese sentences and should be acknowledged when designing studies and interpreting results.

Lastly, the current study used only a set of 12 sentences for the manipulations of local time reversal and modulation-filtering manipulations respectively. While we acknowledge the complexity and challenges in the transcription assessment for Japanese due to the use of multiple writing systems (i.e., Hiragana, Katakana, Kanji, and the Roman alphabet) as described above, future research would benefit from including a larger number of sentences to further validate and extend the findings reported here.

### Conclusion

The current study explored how native Japanese speakers perceive locally time-reversed and modulation-filtered Japanese sentences, following the methodologies used in Ishida et al. (2018). Specifically, this study investigated the effects of language structure and language proficiency on perceptual restoration, as well as the relationship between the duration of local time reversal (ms) and modulation frequency (Hz). In Experiment 1, native Japanese speakers listened to locally time-reversed Japanese sentences in which every local segment of the speech signal was flipped in time at intervals of 10 ms, 30 ms, 50 ms, 70 ms, 90 ms, and 110 ms. In Experiment 2, the same participants listened to modulation-filtered Japanese sentences, where the modulation frequency components were low-pass filtered at cut-off frequencies of 32 Hz, 16 Hz, 8 Hz, 4 Hz, 2 Hz, and 1 Hz. Both experiments showed a decline in intelligibility with increasing degradation, starting from over 95% intelligibility at the lightest degradation. However, there was no direct correspondence between reversed segment length and modulation frequency; the conversion from reversed segment duration (ms) to frequency (Hz) did not capture the critical modulation frequency. Additionally, the intelligibility of L1 Japanese was significantly different from that of L1 English – locally time-reversed speech in L1 Japanese was more intelligible at shorter reversed segment lengths up to 70 ms and less intelligible at longer reversed segment length than L1 English. At the same time, modulation-filtered speech in L1 Japanese was consistently more intelligible than L1 English across all degradation levels. Furthermore, the comparison of L1 Japanese with L2 English (data from Ishida et al., 2018) confirmed the advantage of native language in perceptual restoration within the same individuals (L1 Japanese vs. L2 English with lower intermediate frequency). The higher intelligibility of L1 Japanese under acoustic degradation such as local time reversal and modulation filtering is likely attributable to the characteristics of the Japanese linguistic unit “mora” where vowels appear regularly in CV and V patterns.

## Supplementary Information

Below is the link to the electronic supplementary material.

Supplementary Material 1 (PDF 172 KB)

## Declarations

### Open practices statement

The data for all experiments are available in Open Science Framework (OSF) at https://osf.io/hnfwb/?view_only=37957dd68a8a4c7da991129e2481e07c. Any other information is available upon request from the corresponding author. This study was not preregistered.
