# Supplementary material for: Perceptual restoration of degraded speech: The effects of linguistic structure
Source: Atten Percept Psychophys. 2025 Aug 4;88(2):61. doi: 10.3758/s13414-025-03128-0 (PMC12864291; doi:10.3758/s13414-025-03128-0)
Supplement: Supplementary file 1 — Supplementary Material 1 (PDF 172 KB) [file 13414_2025_3128_MOESM1_ESM.pdf]

Appendix A

一週間ばかりニューヨークを取材した。

ピューンという音と共にすぐ頭上を光が走った。

とにかく事故原因の究明は急いでもらいたい。

話し合いは延々4時間に及んだ。

徐々に沈みつつあるベネツィアの街を救う運動が広がっている。

反政府デモに一般学生や一般市民も合流した。

最初早いテンポで巻き込んでおいて、中盤じっくり見せる。

パジャマとTシャツがめくれて、薄いペチャンコの腹が見えた。

玉ねぎやじゃがいも、キャベツなど、あらゆる野菜を買い込んだ。

夜のディスコパーティーで激しく踊りまくった。

どちらも若いロックファンだけの音楽ではない。

子供たちへのクリスマスプレゼントは、手作りのミュージカルだ。

Appendix B

着用中にダウンやフェザーが飛び出す原因にもなります。

初めてルーブル美術館へ行ったのは 14 年前のことだ。

企業規模別の賃金の格差も少しずつ縮まってきた。

乳母が一年前から入院していて、私が看病をしています。

ハワイやカリフォルニアも良いが、白夜のフィンランドにも行ってみたい。

杜撰さを見落とした、チェックシステムが問題だ。

ダボダボウエアは薄くて、風通しをよくするために考えられた。

ステーキディナーには、アワビのステーキがついている。

白焼きは、わさび醤油であっさりと味わう。

総合的な文化プロデユースだから、全てが目玉なんです。

犬のチャウチャウと大阪弁のチャウチャウをかけている。

お好み食堂にも牛刺しやタンシチューがあった。
